# STAFFING LEVELS AND INJURY-RELATED EMERGENCY DEPARTMENT VISITS IN OHIO

**DOI:** 10.1093/geroni/igad104.1100

**Published:** 2023-12-21

**Authors:** Cassandra Hua, Matt Nelson, Portia Cornell, Elizabeth White, Kali Thomas

**Affiliations:** University of Massachusetts Lowell, Lowell, Massachusetts, United States; Miami University, Oxford, Ohio, United States; Providence VA Medical Center, Providence, Rhode Island, United States; Brown University, Providence, Rhode Island, United States; Johns Hopkins University, Baltimore, Maryland, United States

## Abstract

Assisted living (AL) residents with dementia are at increased risk of injuries compared to residents without dementia. Nursing home research indicates that changes in staffing levels are related to quality, including rate of injuries among residents. We leveraged novel data from a survey administered to AL communities in Ohio from 2007-2015 linked to administrative claims of Medicare beneficiaries with dementia to explore the relationship between staffing levels and injuries among AL residents with dementia. We examined the relationship between changes in each type of staffing level (RN, LPN, and aide hours per resident day [HPRD]) and injury-related emergency department use among AL residents with dementia. We adjusted for demographic characteristics, AL characteristics, and fixed effects for year and AL. Our sample included 13,929 fee-for-service Medicare beneficiaries with dementia (141,749 person-months) from 472 AL communities. Staffing levels increased slightly over time; median aide HPRD were 0.93 in 2007 and 1.10 in 2015. Median RN HPRD were 0.00 in 2007 and 0.04 in 2015. Median LPN HPRD increased from 0.29 to 0.41. Using linear regression modeling, we did not detect a statistically significant relationship between staffing levels (RN, LPN, or aide) and injury-related emergency department visits among AL residents with dementia. Within-AL changes in staffing levels may have no effect on rates of ED visits. However, it is possible that we did not observe enough within-AL variation to detect an association, or that unobserved differences in resident acuity associated with staffing levels biased our finding toward the null.

